# Statistical analysis between 2006 and 2019 and forecast of rabies in cattle from 2020 to 2022 in Tocantins State (Brazil), by using the R Studio software

**DOI:** 10.1017/S0950268822000553

**Published:** 2022-03-29

**Authors:** Alessandro José Ferreira dos Santos, Jardel Martins Ferreira, Francisco Baptista, Bruna Alexandrino, Marco A. Giannoccaro da Silva, José Emerson C. Gomes, José Pereira Veloso Júnior, Raydleno Mateus Tavares, Katyane de Sousa Almeida

**Affiliations:** 1 Center for Advanced Studies in Geoprocessing and Statistics (NEAGE), Agricultural Defense Agency of Tocantins State (Adapec/TO), Rua SE-11, Lote 23, Conj. 3, Setor Central, Palmas, TO 77.020-026, Brazil; 2 Department of Veterinary Epidemiology, Hygiene and Public Health, School of Veterinary Medicine and Animal Science, Federal University of Northern Tocantins (UFNT), Rod. BR 153, km 112, s/n, Post Office Box 132, Araguaína, TO 77.804-970, Brazil; 3 Post-graduate Program in Animal Health and Public Health in the Tropics (PPGSaspt), School of Veterinary Medicine and Animal Science, Federal University of Northern Tocantins (UFNT), Rod. BR 153, km 112, s/n, Post Office Box 132, Araguaína, TO 77.804-970, Brazil; 4 State Program for Control of Rabies in Herbivores (PECRH), Agricultural Defense Agency of Tocantins State (Adapec/TO), Rua SE-11, Lote 23, Conj. 3, Setor Central, Palmas, TO 77.020-026, Brazil

**Keywords:** ARIMA model, bovine rabies, cattle, *Desmodus rotundus* and time series

## Abstract

Rabies in cattle is a viral disease with mandatory notification in Brazil, transmitted by *Desmodus rotundus*, which causes an invariably fatal acute encephalitis. To understand the dynamics of this disease in Tocantins state, Brazil, an analysis of the time series of rabies cases in cattle between 2006 and 2019 was carried out to describe the pattern of its occurrence, aiming to subsidise the Official Veterinary Service (OVS) with relevant information to enable the improvement of control actions provided for in the guidelines of the National Program for the Control of Rabies in Herbivores (NPCRH). The statistical analyses of the time series under study were performed using the R Studio software, version 1.1.463, in which the existence of trend, cyclicality and seasonality of rabies cases in cattle was assessed. These analyses showed that this disease is endemic in Tocantins state, with epidemic outbreaks that can occur every 3 or 4 years, without a seasonality pattern. The autoregressive integrated by moving average (ARIMA(4,1,4)) model predicted the approximate occurrence of 38 rabies cases in cattle in 2022 and all monthly records of this disease remained within the predicted confidence interval (95% CI) in 2020 and 2021, demonstrating it has a good predictive capacity and allowing OVS to intervene in the present processes to achieve better control of this disease.

## Introduction

Rabies is an infectious disease caused by a virus belonging to the genus *Lyssavirus*, family Rhabdoviridae, consisting of single-chain ribonucleic acid. In Brazil, it is a mandatory reporting disease that affects the central nervous system of people and all species of domestic and wild mammals. It is one of the main zoonoses in the world, with important implications for public health due to the high economic and social cost related to the damage caused in livestock, as it is an invariably fatal disease [1–3].

In Brazil, *Desmodus rotundus* is the main transmitter of rabies to cattle, causing incoordination, paralysis and hypoesthesia of pelvic limbs, and the diagnosis is carried out in an official or accredited laboratory using a direct immunofluorescence test associated with intracerebral inoculation in mice or cell culture. The combat strategies of the Brazilian Program for Rabies Control in Herbivores are based on epidemiological surveillance, vaccination of domestic herbivores, population control of *D. rotundus*, health education and control of outbreaks [1, 4–6].

Cattle herd in Tocantins state increased 9.14% from 2009 to 2019, being the 11th largest herd in Brazil in 2019, with 8 300 111 animals, representing 3.88% of the national herd, behind of Pará and Rondônia states in the North Region, which had 20 510 169 and 13 973 714 animals that same year, respectively. In the Tocantins economy, the agricultural sector represents, on average, 13.1% of the added value of the gross domestic product, which was estimated at R$35.6 million in 2018 [7, 8].

Considering the economic relevance of cattle farming in Tocantins state, knowledge about the dynamics of rabies in cattle is of paramount importance to identify the pattern of its occurrence over time and subsidise the Official Veterinary Service (OVS) with information to guide the implementation of sanitary measures to control this zoonosis. Thus, this study aimed to describe the pattern of occurrence of cattle rabies in Tocantins state by analysing the time series from January 2006 to December 2019, as well as assess the most appropriate predictive model for making predictions of cases of this disease from 2020 to 2022.

## Material and methods

### Location of the study area

**Fig. 1.**
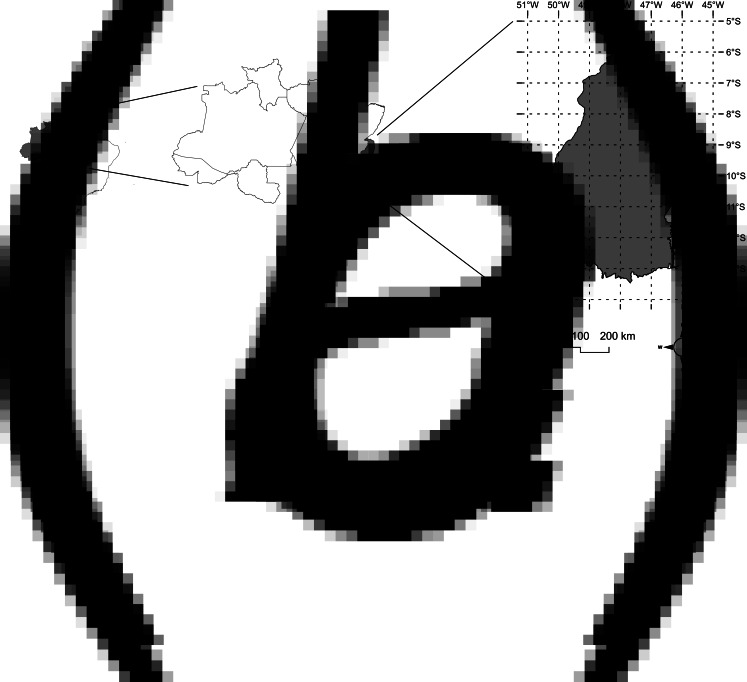
Geographic location of Tocantins state. (a) Brazil, (b) North Region and (c) Tocantins.

The study was carried out in Tocantins state, located in North Region of Brazil, between the meridians 45° and 51° west longitude and the parallels 5° and 14° south latitude (Fig. 1). This state is composed of 139 municipalities, with a total area of 277 620.914 km^2^. It has a semi-humid climate divided into two zones, Tropical Equatorial Zone in the north of the state and Tropical Central Brazil in the south, characterised by two well-defined seasons throughout the year due to the proximity to the Equator Line, with a rainy season in the summer, from October to April, and a dry season in the winter, from May to September. The temperature varies from 25 to 40 °C, with annual rainfall above 1500 mm [9].

### Data collection

The pattern of occurrence of cattle rabies in Tocantins state was analysed from the monthly data for the period between 2006 and 2017, which were extracted from the interactive database panel of the National Zoosanitary Information System (SIZ) of the Ministry of Agriculture, Livestock and Food Supply (MAPA), and those referring to the period between 2018 and 2021 were extracted from the platform of the State Program for the Control of Rabies in Herbivores (SisPECRH) of the Agricultural Defense Agency of Tocantins State (ADAPEC/TO). A case of rabies is understood as a sick or infected animal, with a confirmed diagnosis of the disease [10]. The data were consolidated in a Microsoft Excel 2010^®^ spreadsheet saved in the CSV (comma-separated values) format. Subsequently, the data were transformed into a time series using the ‘ts()’ function native to the R Studio software, version 1.1.463.

### Statistical analysis and forecast

The forecasting from the time series of rabies cases in cattle and the respective statistical analyses were performed using the R Studio software. A time series is represented by the combination of the trend-cycle, seasonality and error components and can be described as follows [11]:
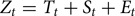
where *Z_t_* is the data observed in period *t*, *T_t_* is the trend-cycle component in period *t*, *S_t_* is the seasonal component in period *t* and *E_t_* represents the part not captured in the model in period *t*, called error or random residual.

The time series (*Z_t_*) of rabies cases in cattle was decomposed using the function ‘decompose()’, native to the R Studio software so that its three components could be viewed individually. The trend-cycle component (*T_t_*) was analysed by linear regression using the function ‘lm()’, native to the R Studio software. The functions ‘efp()’ and ‘sctest ()’, belonging to the statistical package ‘strucchange’ [12], were used to verify the existence of structural breaks in the process of the time series under study by statistical method ‘OLS-CUSUM’. The occurrence of epidemic cycles of rabies was assessed using the function ‘filter()’ [13]. The existence of the seasonal component (*S_t_*) in the time series was verified using the functions ‘qs()’, ‘sesas()’ and ‘series()’, belonging to the statistical package ‘seasonal’ of the program ‘X-13 ARIMA- SEATS’ [14].

The autoregressive integrated by moving average (ARIMA) model was defined based on the Box and Jenkins methodology for stationary time series, briefly described below [11]:

- *Specification*: analysis of the general class of ARIMA(*p*,*d*,*q*)(*P*,*D*,*Q*) structures, where *p* is the order of the non-seasonal autoregressive polynomial, *P* is the order of the seasonal autoregressive polynomial, *d* is the order of non-seasonal differentiation, *D* is the order of seasonal differentiation, *q* is the order of the non-seasonal moving average polynomial and *Q* is the order of the seasonal moving average polynomial.
- *Identification*: the *p*,*q* values were defined based on the autocorrelation function (ACF) and the partial autocorrelation function (PACF). An ARIMA(*p*,*d*,*q*) process is an autoregressive and moving average (ARMA) differentiated *d* times until it becomes stationary.
- *Estimation*: the parameters of the identified model were statistically tested for significance with the function ‘*t*_test()’, belonging to the package BETS [15].
- *Diagnosis*: an analysis of residuals and verification tests (Ljung–Box) were carried out to assess whether the suggested model is suitable for predictions using the functions ‘tsdiag()’, ‘Box.test()’ and ‘shapiro.test()’, which are native to the R Studio software.
- *Forecast*: the function ‘forecast()’ of the statistical package forecast [16] was used to forecast rabies cases in cattle.

The accuracy of the ARIMA predictive model in future observations was assessed by the cross-validation of Holdout, aiming to assess the generalisation capacity of the model. For this, the function ‘window()’, native to the R software, was used. In this case, the data from 2006 to 2017 were used as training and the data from 2018 and 2019 were used as a test, considering the value of Theil's *U*-statistic, whose result, according to Ehlers [17], must be lower than one for the model to have a good predictive capacity.

## Results

**Fig. 2.**
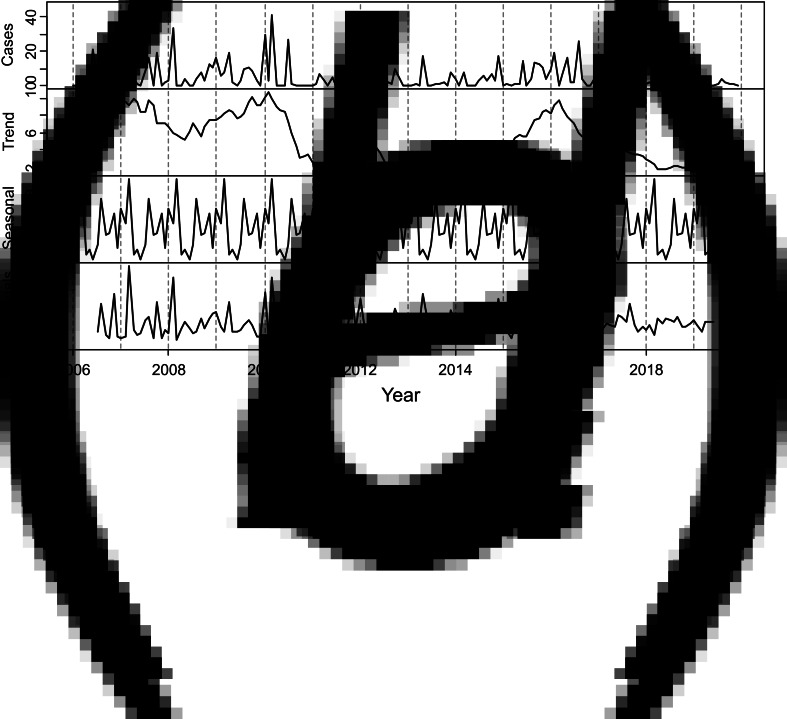
Decomposition of the time series of cattle rabies cases in Tocantins state, Brazil, 2006–2019. (a) Original series, (b) trend-cycle, (c) seasonal and (d) random error.

**Fig. 3.**
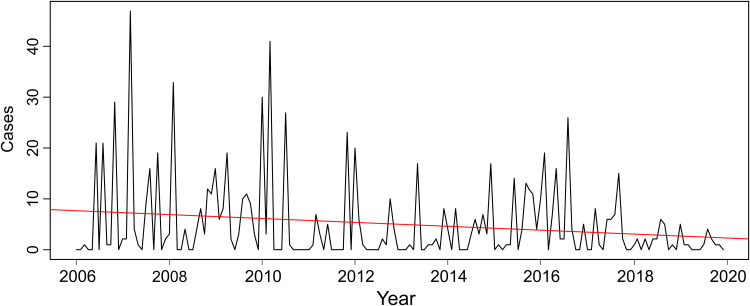
Linear regression test for the time series of cattle rabies cases in Tocantins state, Brazil, 2006–2019.

**Fig. 4.**
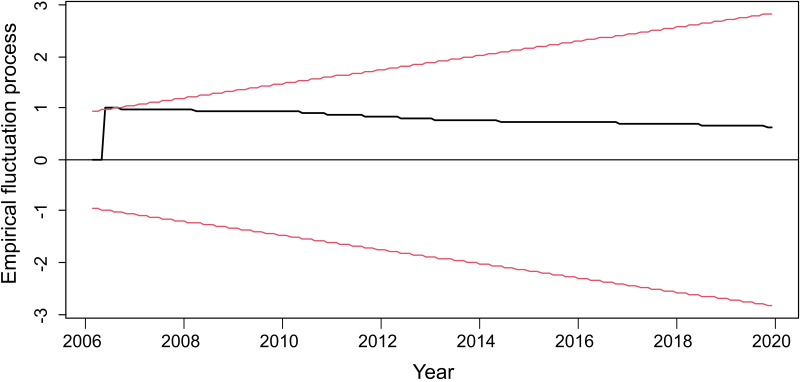
Spectral of the empirical fluctuation process of the time series of cattle rabies cases in Tocantins state, Brazil, 2006–2019.

**Fig. 5.**
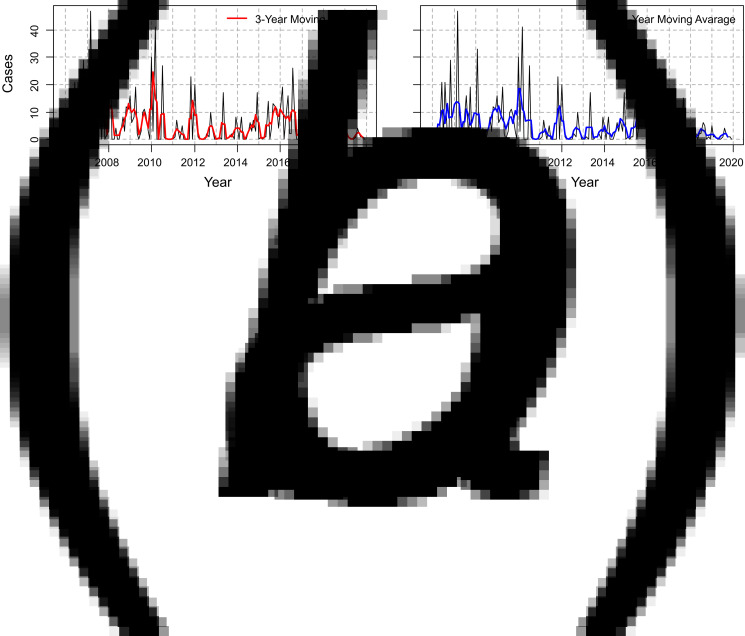
Epidemic cycle of cattle rabies in Tocantins state, Brazil, 2006–2019: (a) estimate of epidemic occurrence every 3 years; (b) estimate of epidemic occurrence every 4 years.

**Fig. 6.**
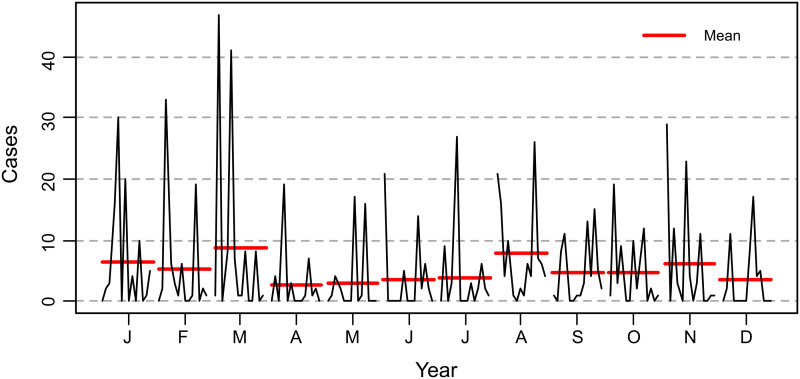
Monthly average of cattle rabies cases in Tocantins state, Brazil, 2006–2019.

The data transformed into time series of cattle rabies cases in Tocantins state (Fig. 2a) were broken down into the following components: trend-cycle (*T_t_*) (Fig. 2b), seasonal (*S_t_*) (Fig. 2c) and error/residual (*E_t_*) (Fig. 2d). A total of 841 rabies cases in cattle were observed in Tocantins state between 2006 and 2019, showing a declining trend, as demonstrated by the linear regression test (*y* = − 0.3886*x* + 787.2497, *P* < 0.05, *R*^2^ = 0.0377) (Fig. 3) and the structural break observed in 2006 in the studied time series (*S* = 0.96122, *P* < 0.05) (Fig. 4). However, epidemic outbreaks of rabies seem to occur with cyclicity of 3–4 years in Tocantins state (Fig. 5a–b). The seasonality analysis showed no statistical significance (*P* > 0.05) for the original series (qsori), that is, rabies cases in cattle do not occur seasonally, but a lower average occurrence of these cases was observed from April to June (Fig. 6).

**Fig. 7.**
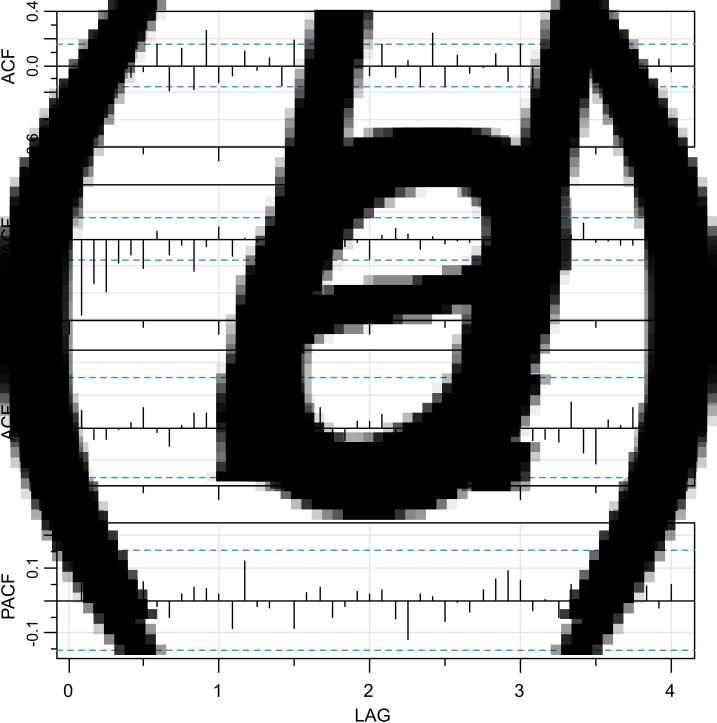
Correlograms of the time series of cattle rabies cases in Tocantins state, Brazil, 2006–1019: (a) correlogram of the autocorrelation function (ACF); (b) correlogram of the partial autocorrelation function (PACF); (c) correlogram of residuals of the autocorrelation function (ACF); (d) correlogram of residuals of the partial autocorrelation function (PACF).

The Zivot–Andrews test of unitary root or stationarity sowed the necessity of the original time series of cattle rabies cases in Tocantins state to go through a differentiation process to obtain a stationary series. Therefore, the non-seasonal differentiation order (*d*) was defined as being equal to one. The ACF and PACF correlograms provided an indication of the specification and identification of the polynomials (*p*) and (*q*) of the ARIMA model (Fig. 7a–b). Thus, the ARIMA(4,1,4) model was estimated, which was confirmed by the significance test and the analysis of the correlograms of residuals of the model in ACF and PACF (Fig. 7c–d), as all the lags were within the limit of the confidence interval. The Ljung–Box diagnosis for the estimated model showed no linear correlation (*χ*^2^ = 28.045, df = 23, *P* > 0.05) and the Shapiro–Wilk test showed no residual normality (*W* = 0.82443, *P* < 0.05).

The predictive accuracy of the ARIMA(4,1,4) model was assessed from the training and test data of the studied time series, with Theil's *U*-statistic value equal to zero. This model predicted the occurrence of approximately 38 rabies cases in cattle in Tocantins state in 2022 (Table 1). Also, all monthly records of this disease remained within the predicted confidence interval (95% CI) in 2020 and 2021 (Table 1).

**Table 1.** Prediction of cattle rabies cases in Tocantins state, Brazil, from 2020 to 2022 using the predictive model ARIMA(4,1,4)

Month	2020			2021			2022		
	Predicted^a^	Recorded	95% CI^a^	Predicted^a^	Recorded	95% CI^a^	Predicted^a^	Recorded	95% CI^a^
Jan.	3	0	0–18	3	3	0–20	3	N/A	0–20
Feb.	1	0	0–16	3	2	0–20	3	N/A	0–20
Mar.	5	0	0–21	2	3	0–19	4	N/A	0–20
Apr.	0	0	0–16	4	3	0–20	3	N/A	0–20
May	3	0	0–19	3	0	0–19	3	N/A	0–20
June	4	1	0–19	3	3	0–19	3	N/A	0–20
July	1	2	0–17	4	1	0–20	3	N/A	0–20
Aug.	4	2	0–20	3	2	0–19	4	N/A	0–19
Sept.	2	0	0–18	3	1	0–20	3	N/A	0–20
Oct.	2	2	0–18	3	3	0–20	3	N/A	0–20
Nov.	4	0	0–20	3	0	0–19	3	N/A	0–19
Dec.	2	0	0–18	4	2	0–20	3	N/A	0–20
Total	31	7	–	38	23	–	38	N/A	–

N/A, entry not available.

a Rounded values.

## Discussion

Cattle rabies in Tocantins state is endemic and, according to Barros *et al*. [1], this enzootic stability occurs due to favourable climate conditions, the existence of natural and artificial shelters for haematophagous bats, and distribution of the cattle population in a given region. Despite the trend of declining casuistry (Figs 2b and 3), an increase in the number of these cases was predicted for 2021 and 2022 (Table 1) since, according to the time series analysis under study, rabies presents a cyclical characteristic in the occurrence of epidemic outbreaks every 3 or 4 years (Fig. 5a–b). This observation is similar to that found by Oliveira *et al*. [18], who assessed the frequency of rabies in herbivores and humans from 1999 to 2010. However, Barros *et al*. [1] found that these outbreaks occur with a periodicity of approximately 7 years due to the higher number of infected haematophagous animals during peak cases in herbivores. Moreover, the epidemic cyclicality can be affected by sanitary measures, such as vaccination of the cattle herd and population control of the haematophagous bat *D. rotundus*, but according to Barros *et al*. [1], periods of disease decline may be related to the death of infected bats and the cyclical disappearance of rabies does not represent its effective control.

Barros *et al*. [1] also observed a reduction in the number of rabies cattle cases in the Midwest and Southeast regions from September to March related to the biological cycle of the haematophagous bat mainly due to the dispute between males for females that occurs in the spring (September to December), but this observation contradicts the results found in this study, as April to June present the lowest average occurrence of cattle rabies in Tocantins state (Fig. 6). It shows the importance of carrying out further studies to determine whether the characteristics of the biological cycle of the bat *D. rotundus*, especially the reproductive cycle, are related to the regional climate conditions since no clear distinction between the four seasons is observed in Tocantins state.

The studied time series showed no seasonality in the occurrence of rabies cases in cattle, corroborating with Gomes and Monteiro [19], who evaluated the distribution of cattle rabies in São Paulo state, and Lopes *et al*. [3], who studied the time series of rabies cases in Minas Gerais state. However, the lowest averages of occurrence of rabies from April to June allow the OVS to institute a specific state calendar of rabies vaccination in May, applying the booster dose to the first vaccinated animals after 30 days and annual revaccination of the entire herd, especially in municipalities where rabies has a higher incidence. Considering that the immunisation window for rabies vaccination is 21 days, this measure would allow a higher number of animals to be immunised before August, the period in which the highest occurrence of cattle rabies cases in Tocantins state begins (Fig. 6).

According to Tizard [20], vaccines must be administered before the period when outbreaks of any disease are expected. This author also reported that the prophylactic vaccination strategy significantly reduces the potential for a major epidemic by decreasing the size of the susceptible population, and the effectiveness of this procedure can be improved by identifying high-risk herds and ensuring that they have been protected before an outbreak has occurred. Thus, May seems to be the most suitable month to adopt the prophylactic vaccination strategy against cattle rabies in Tocantins state, but the success of this strategy depends on the awareness of farmers through health education, as well as association with other control measures adopted by OVS, such as improvement of epidemiological surveillance, population control of the haematophagous bat *D. rotundus* and immediate attendance to the notification of suspected outbreaks of the disease.

The ARIMA(4,1,4) model was adequate to make predictions of rabies cases in Tocantins state, as it met the criterion of the absence of residual autocorrelation. Despite the Shapiro–Wilk test demonstrating the absence of normal residuals, Barros *et al*. [11] affirmed that this is not a necessary characteristic in the diagnosis of ARIMA models and, therefore, it does not decrease the predictive capacity of the model. The identification is the critical stage of constructing ARIMA models. According to Morettin and Toloi [21], several researchers can identify different models for the same time series, but the forecasts are obtained with higher precision than other forecasting methods due to the small number of parameters.

The ARIMA(4,1,4) model predicted the approximate occurrence of 38 rabies cases in cattle in 2022 and all monthly records of this disease remained within the predicted confidence interval (95% CI) in 2020 and 2021 (Table 1), demonstrating that the model has good short-term predictive capacity. However, some factors may limit the predictive capacity of this technique, such as the absence of notification to OVS of the occurrence of rabies by farmers and the number of samples collected for diagnosis of a suspected outbreak of neurological syndrome.

According to Antunes and Cardoso [22], the need to predict the future in epidemiological studies and intervene in the current processes based on this information is essential for allowing the planning of actions to combat diseases, as the reduction of health indicators of a given population, for instance, incidence and prevalence, depends on the effectiveness of the interventions. Thus, this type of study allows the targeting of financial and human resources to improve control actions provided for in the National Program for the Control of Rabies in Herbivores (NPCRH) guidelines. Despite this study was conducted with data on the occurrence of cattle rabies in Tocantins state, this method can be used to analyse time series of other infectious diseases, and predict the emergence of new cases of these diseases in any geographic regions.

## Conclusion

Rabies in cattle is endemic in Tocantins state, with a cyclical pattern of epidemic outbreaks that seems to be repeated every 3 or 4 years, but with no seasonality and periods of less occurrence between April and June, being May the month indicated for the prophylactic vaccination of the herd, with an emphasis on municipalities with the highest incidence of the disease. Research is necessary to determine the biological cycle of the bat *D. rotundus* in Tocantins state, mainly on factors inherent to reproductive characteristics, and correlate them with the pattern of occurrence of this disease in cattle. Finally, the ARIMA predictive model was adequate to predict from the studied time series, demonstrating to be a useful tool in decision-making and planning actions to combat rabies in cattle not only in Tocantins state, but also in other geographical regions where paralytic rabies is a problem too.

## Data Availability

All materials needed to replicate the findings of the article are available as Supplementary Materials.

## Supplementary material

For supplementary material accompanying this paper visit http://doi.org/10.1017/S0950268822000553. click here to view supplementary material
